# A New Remedy for Sea-Sickness

**Published:** 1881-10

**Authors:** T. S. Hopkins

**Affiliations:** Thomasville, Ga.


### A NEW REMEDY FOR SEA-SICKNESS.

By T. S. HOPKINS, M.D., Thomasville, Ga.

I desire to call the attention of all who "go down to the sea in ships," to a new remedy for sea-sickness. In a recent voyage from Savannah to Philadelphia, by steamship, I found the following prescription very successful:

℞—Potassii bromidi, - - - ℥ss.

Ergotæ ext. fld. (Squibb) - - ℥ss.

Aquæ - - - - ad. ℥vj.

M. S.—Tablespoonful; repeated, if necessary, three times daily.

The ergot did the work. The bromides alone have always failed me. The well known power of ergot in contracting the arterioles induced me to try it. The prescription was being used by me in a case of insomnia, symptomatic of what I considered a very well marked case of cerebrasthenia. A single dose of the mixture relieved one of the worst cases on the ship. It is preventive as well as curative.

